# Long-term memory guides resource allocation in working memory

**DOI:** 10.1038/s41598-020-79108-1

**Published:** 2020-12-17

**Authors:** Allison L. Bruning, Jarrod A. Lewis-Peacock

**Affiliations:** Department of Psychology, Center for Learning and Memory, University of Texas at Austin, 108 E Dean Keeton St, Stop A8000, Austin, TX 78712 USA

**Keywords:** Human behaviour, Attention

## Abstract

Working memory capacity is incredibly limited and thus it is important to use this resource wisely. Prior knowledge in long-term memory can aid in efficient encoding of information by allowing for the prioritization of novel stimuli over familiar ones. Here we used a full-report procedure in a visual working memory paradigm, where participants reported the location of six colored circles in any order, to examine the influence of prior information on resource allocation in working memory. Participants learned that one of the items appeared in a restricted range of locations, whereas the remaining items could appear in any location. We found that participants’ memory performance benefited from learning this prior information. Specifically, response precision increased for all items when prior information was available for one of the items. Responses for both familiar and novel items were systematically ordered from highest to lowest precision. Participants tended to report the familiar item in the second half of the six responses and did so with greater precision than for novel items. Moreover, novel items that appeared near the center of the prior location were reported with worse precision than novel items that appeared elsewhere. This shows that people strategically allocated working memory resources by ignoring information that appeared in predictable locations and prioritizing the encoding of information that appeared in unpredictable locations. Together these findings demonstrate that people rely on long-term memory not only for remembering familiar items, but also for the strategic allocation of their limited capacity working memory resources.

## Introduction

Working memory (WM), our ability to maintain and manipulate information for goal-directed behavior, is essential to accomplishing everyday tasks. As we navigate our environments, we must maintain current goals and encode new information, all while evaluating whether action can be taken to accomplish these goals. The demands of WM are daunting compared to its limited capacity^1,2^, yet cognitively healthy people routinely perform complex, flexible, and adaptive behaviors. This is made possible in part by selective and strategic allocation of WM resources. For example, our behavioral goals dictate what information in our environment is most important. Not all information is equally relevant to the current goal. When waiting at an intersection, the traffic light in front of your vehicle is far more relevant in determining when it is permissible to cross the intersection than the traffic light for the orthogonal direction. You can prioritize the most relevant traffic light to determine a safe time to move with fewer errors than if you were to instead prioritize the other less-relevant traffic lights. This optimizes the use of WM resources by narrowing the breadth of what to encode with little cost to decision accuracy. The flexible nature of WM also allows for the unequal distribution of resources, whereby different items can receive varying amounts of representation^3,4^. In the traffic intersection example, you could allocate the majority of WM resources to the nearest traffic light, but also attend to the cross traffic, the other traffic lights, and the crosswalk sign to supplement your decision process about when it is safe to drive through the intersection. In the lab, participants in a visual WM task prioritize the encoding of behaviorally relevant items^5–7^ or object features^8–10^ over less relevant items or features. Furthermore, behavioral performance in a visual WM task is more accurately modeled when prioritization is taken into account as opposed to the stimulus set size^11^.

Information already encoded in WM can also influence the relative importance of information in our environment. Unconsciously, information in WM can influence reaction times in visual search tasks through attentional capture, where subjects are quicker to find features held as an active representation in WM^12,13^. Consciously, knowing what is in WM and the quality of that information, i.e. uncertainty^14^, can determine what information should be prioritized. If knowledge of two items is needed to complete a task, but one item is imprecisely encoded in WM, that item could be prioritized to gain a greater amount of information or reward. Previous work has shown that participants can and do make use of the uncertainty of WM representations to optimize eye movements^15^ and reward decisions^16^ . This effect can even be seen in visual search patterns, where participants are slower to respond to stimuli in locations that were recently attended, i.e. inhibition of return^17^.

Information stored in long-term memory (LTM) can also facilitate encoding in WM. Knowledge in LTM can increase the amount of information encoded through compression or chunking, where a “smaller memory file” is held in WM that acts as a pointer to the full representation held in LTM^18–20^. The process of compression may look similar to disambiguation in more naturalistic settings in which an object is recognized based on previous experience^21–23^. Familiar items are remembered at a greater capacity^24,25^ and are processed faster^26^ than less familiar items. Beyond compression, previous experience can guide WM to more probable areas where relevant information will be^27,28^. This information in LTM only comes online when relevant, providing facilitation without interference^29,30^. It is also possible that LTM information could guide the allocation of WM resources *away* from probable areas, and instead towards less probable areas where novel relevant information could be found.

**Figure 1:**
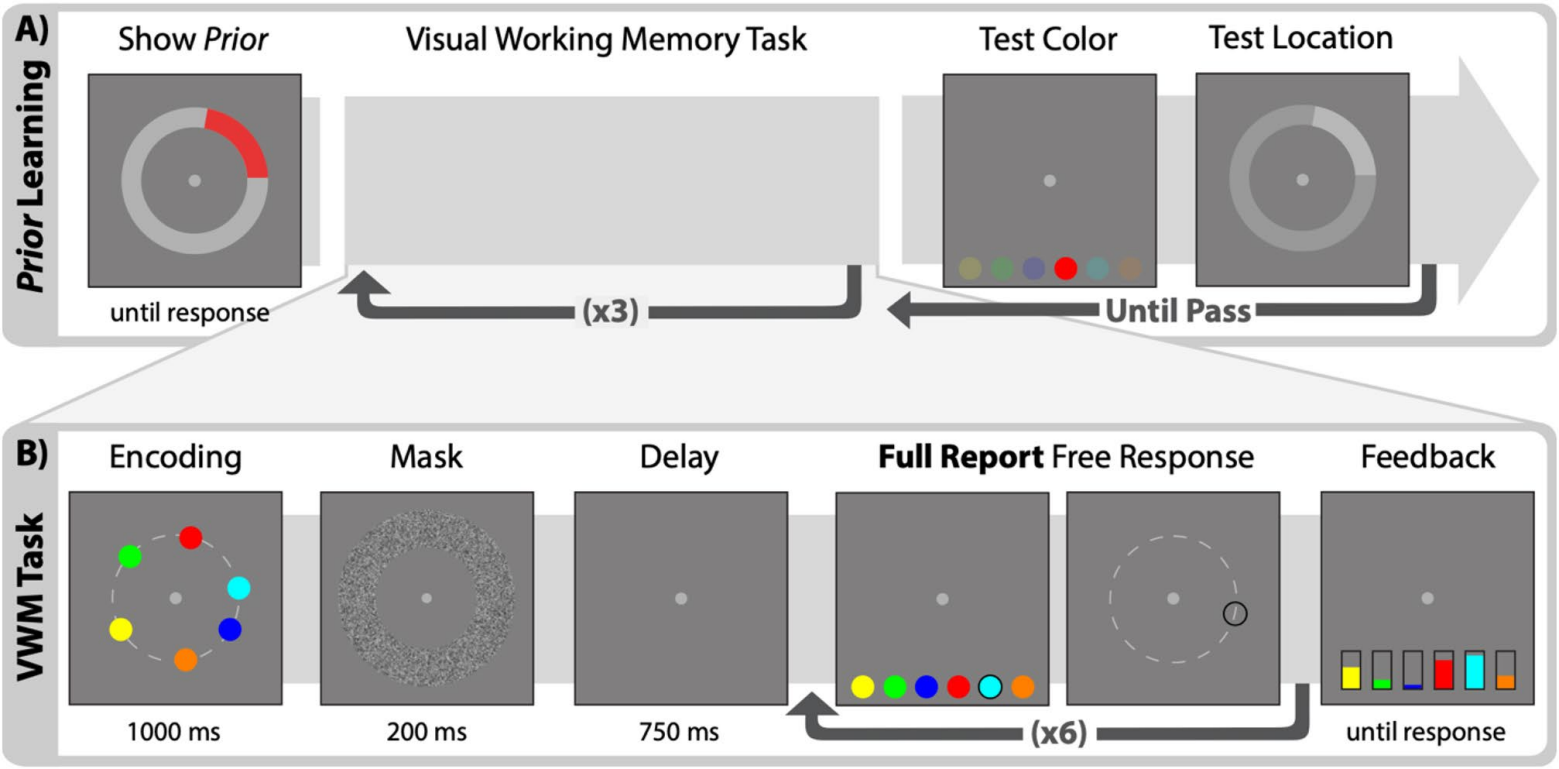
Task design. (**A**) Explicit learning for prior information. Participants were shown both the color and location of the prior distribution (width = 80°). Here the prior is red and centered at 45°. This information remained constant throughout the experiment. After three practice trials, participants were tested on both the color and location of the prior (correct responses shown at full opacity). (**B**) Visual working memory task. For each trial, participants were tasked to report the location of all six colors in any order they chose. All six colors were used in every trial. Items appeared about a fixed radius where the location was determined by a random angle. The location of the familiar item was determined by a von Mises distribution while the remaining five colors were determined by a uniform distribution.

In the present study, we sought to evaluate the potential benefits and costs when participants use prior information in LTM to strategically allocate the limited resources of WM. To test this, we used a delayed-estimation task with a full-report procedure. Participants were required to report spatial location for all six colored circles presented on each trial (Fig. 1). To incorporate prior information into this task, the location of one of the six colors was determined by a von Mises distribution (familiar item), while the remaining five colors were determined by a uniform distribution (novel items). Because the focus of these experiments was not whether statistical regularities can be learned, participants were explicitly shown this information to avoid this time-consuming process. We used model-free statistical analyses to quantify the accuracy of responses for the spatial location of all six items on every trial. We hypothesized that participants could improve performance on this task by prioritizing the encoding of novel items in WM and relying on LTM to report the location of the familiar item. However, such prioritization could lead to systematic costs for items that appear near the de-prioritized familiar item, despite the overall improvement in performance. Our analyses used response order and estimates of response precision to evaluate the cost/benefit tradeoffs of prioritizing novel information in WM.

## Results

**Figure 2:**
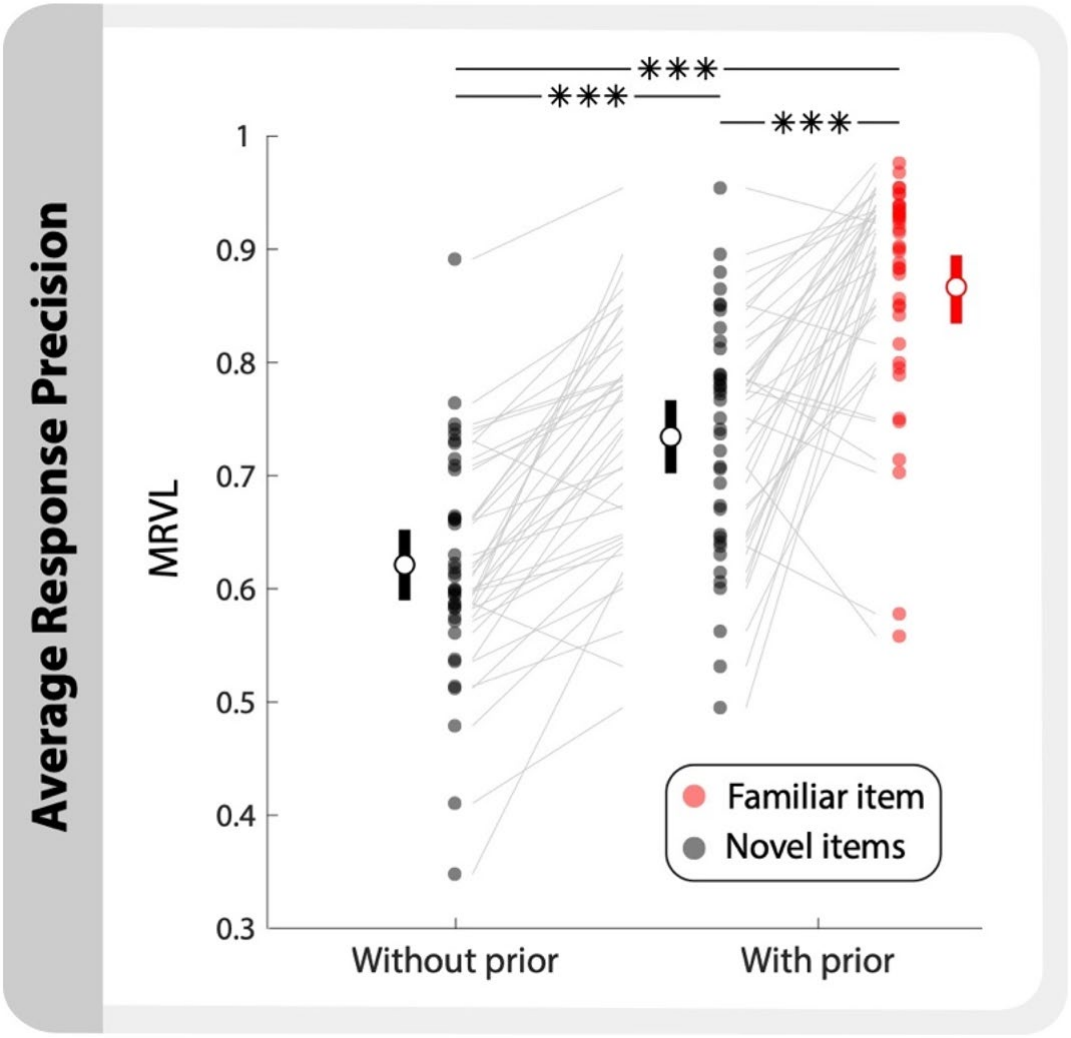
Greater response precision when prior information is known. Response precision (MRVL) with and without the prior information for both the familiar and novel items. White circles indicate group mean; bars indicate bootstrapped 95% CIs. Dots and lines represent individual participants. (*** *p* < 0.0001).

This study was split into two experiments. Both experiments had the same main task (described in Fig. 1), and only differed in the first block of trials. Participants from experiment 1 (n = 39) were not shown the prior information and had no statistical regularities until after the first block of trials. Therefore, all items in the first block were novel items, and the subsequent seven blocks had one familiar item and five novel items on each trial (Fig. 2). Participants from experiment 2 (n = 23) learned the prior information before the first block of trials and therefore all eight blocks contained one familiar item and five novel items. Excluding Fig. 2, we combined data from both experiments to increase statistical power. No effect was found from experiment group excluding two analyses which are noted in the results below.

### Response precision increases with prior information

To examine the effect of the prior information on response precision, we compared responses from experiment 1 made with and without the prior information present. Without prior information, all six items were considered novel items. Error was defined as the angular distance between the participant’s response and the true location. To summarize across the error distributions, we used mean resultant vector length (MRVL) as a metric of precision^31^, where MRVL ranges from 0 to 1 indicating either no information or perfect information about the target, respectively. The familiar item data only included responses to that item, whereas the novel items data included responses to all other items. MRVL increased significantly once the prior information was introduced for both item types: familiar item (*t*(38) = 10.81, *p*_*(Bon)*_ < 0.0001, *d* = 2.44, *α* = 0.017) and novel items (*t*(38) = 8.59, *p*_*(Bon)*_ < 0.0001, *d* = 1.09, *α* = 0.017) (Fig. 2). Additionally, the increase in precision was greater for the familiar item (*t*(38) = 6.34, *p*_*(Bon)*_ < 0.0001, *d* = 1.28, *α* = 0.017).

To test whether this increase was only due to a practice effect, the same analysis was done on participants from experiment 2 where the familiar item was present for all blocks of trials. Responses in the first block had significantly lower precision than the remaining blocks of trials (*t*(22) = 2.48, *p* = 0.021, *d* = 0.48) indicating a practice effect was present. To control for this, we compared precision from the first block of trials between the two experiments. Even without a practice effect, precision with prior information was greater than precision without prior information (ind. *t*(60) = 3.78, *p* = 0.0004, *d* = 0.99). For the remaining analyses participants were collapsed across both experiments.

### Familiar item responses are more stable than novel item responses

**Figure 3:**
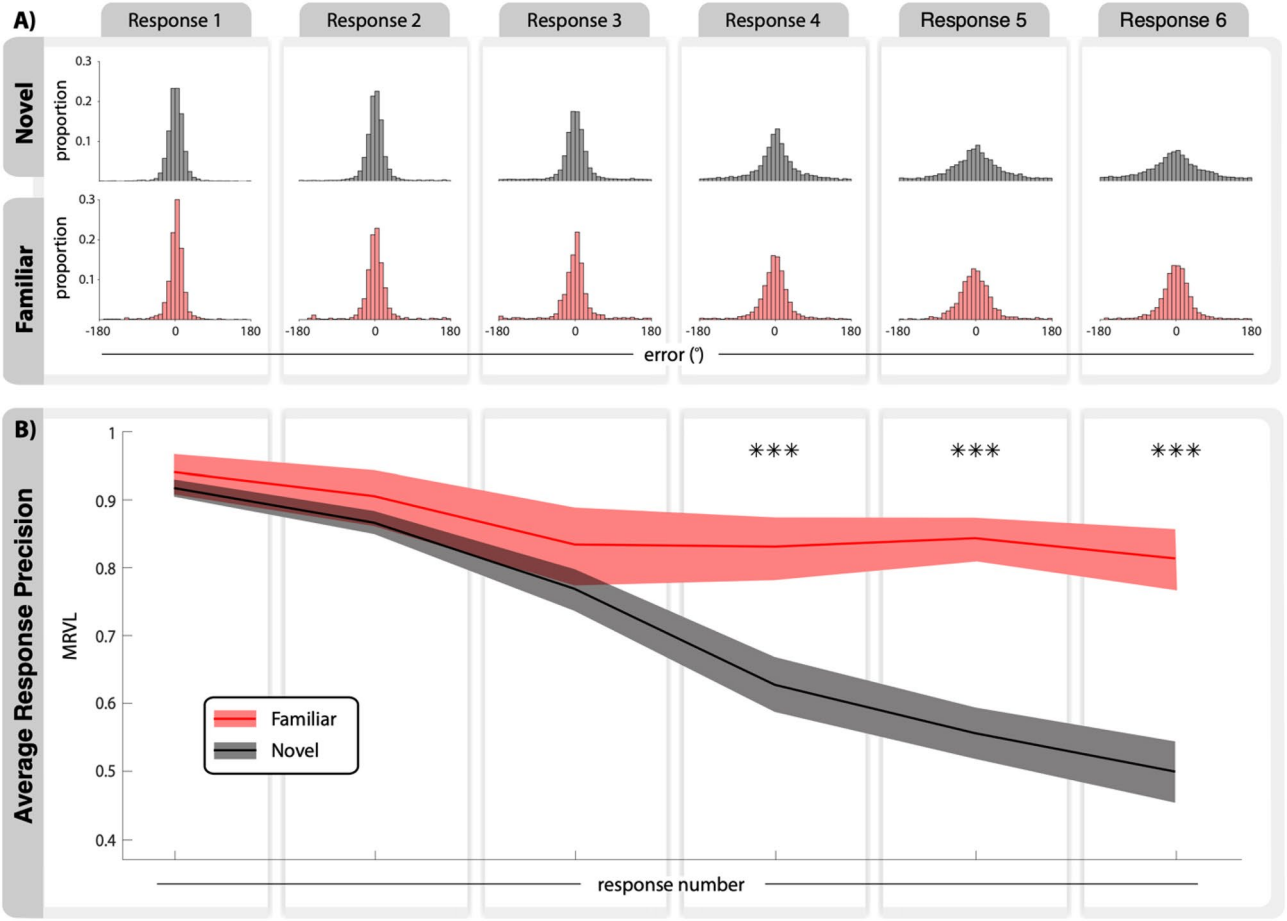
Later responses have greater precision when reporting familiar items. (**A**) Aggregate error distributions across all participants split by item type and response number. (**B**) Precision (MRVL) for items at each response number. Line indicates group mean and shaded area indicates bootstrapped 95% CIs (*** *p* < 0.0001).

Next, we examined the effect of item type (familiar or novel) and response number (1st-6th) on response precision. The error distributions for every response combination are shown in Fig. 3A. We found a significant main effect of item type (*F*(1, 61) = 94.63, *p* < 0.0001, η_p_^2^ = 0.61) and response number (*F*(5, 305) = 144.25, *p* < 0.0001, η_p_^2^ = 0.70), as well as a significant interaction of these two factors (*F*(5, 305) = 41.08, *p* < 0.0001, η_p_^2^ = 0.40) (Fig. 3B). Specifically, precision for familiar and novel items only differed for the last three of six total responses (response 4: *t*(60) = 7.49, *p*_*(Bon)*_ < 0.0001, *d* = 1.14, *α* = 0.0083; response 5: *t*(59) = 11.71, *d* = 1.95, *α* = 0.0083, *p*_*(Bon)*_ < 0.0001; response 6: *t*(60) = 9.38, *p*_*(Bon)*_ < 0.0001, *d* = 1.60, *α* = 0.0083). The influence of response number had a negative linear relationship for both the familiar item (*β* = -0.024, *p* < 0.0001, *R*^2^_*(adj)*_ = 0.06) and novel items (*β* = -0.087, *p* < 0.0001, *R*^2^_*(adj)*_ = 0.58) where earlier responses had greater precision than later responses.

### Response order is non-uniform for the familiar item

**Figure 4:**
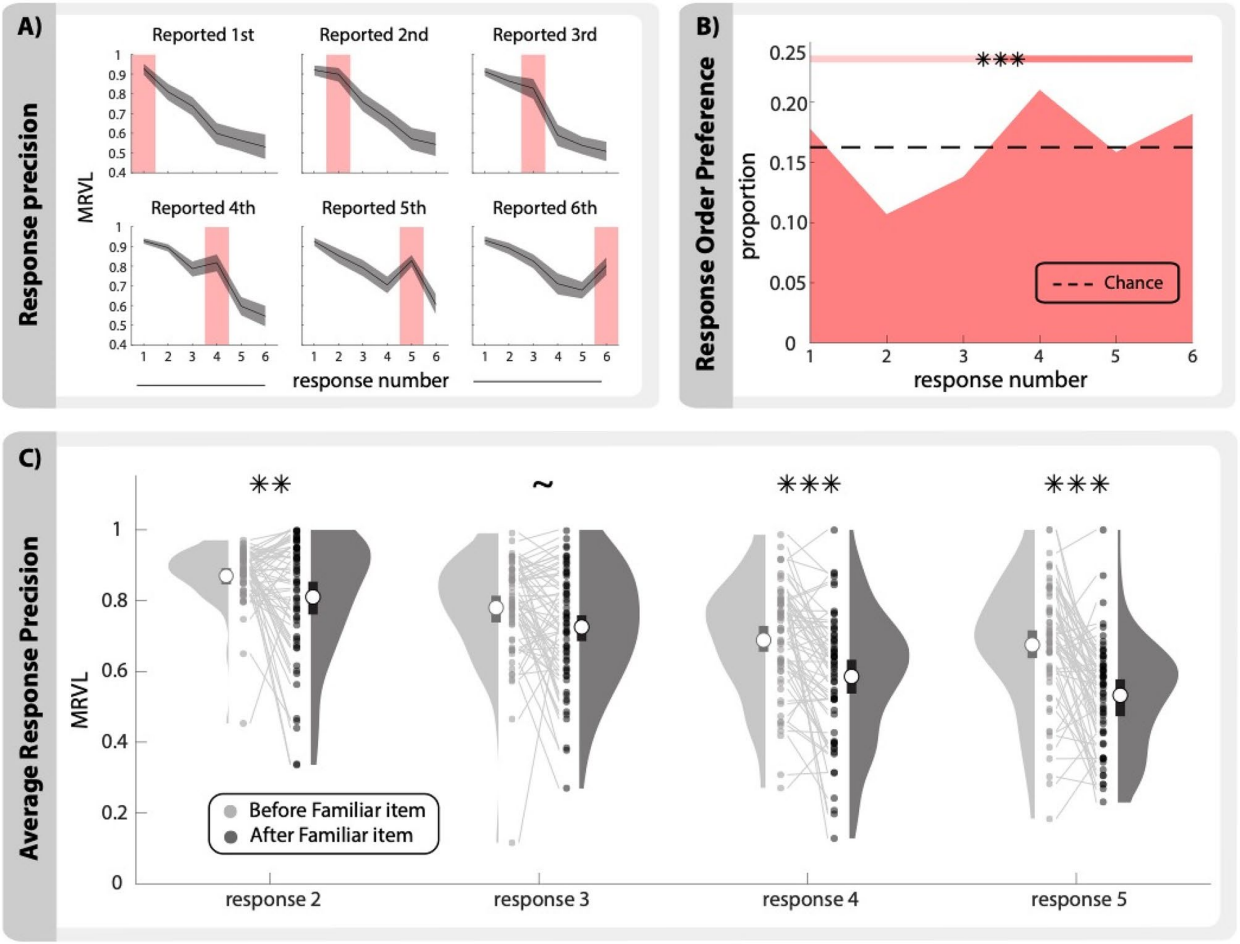
Bias in response order. (**A**) Precision for all items split by when the familiar item was reported. Red shaded area indicates responses to the familiar item. (**B**) Proportion of responses to familiar items at each response number where chance is 1/6 and shown by the dashed line. Colored area with significant markers indicates bins for binomial test. (**C**) Response precision (MRVL) for novel items at each response number split by relative order with the familiar item. Response number indicates when the novel item was reported while *before* and *after* indicate when the familiar item was reported (e.g. response 2 before would be all novel item responses reported second when the prior item was reported third or later). Only responses 2–5 are shown because responses 1 and 6 do not have both before and after responses. White circles indicate group means; bars indicate bootstrapped 95% CIs. Dots and connecting lines represent individual participants. (~ *p* < 0.05, ** *p* < 0.01, *** p < 0.0001).

Within trials, participants did not always report the familiar item in order of precision along with the novel items (i.e. reporting the familiar item 5th or 6th would typically follow a lower precision novel item) (Fig. 4A). We examined whether participants were more likely to report the familiar item at any particular response position and found that these responses were non-uniform (*X*^2^(5, *N* = 8351) = 379.58, *p* < 0.0001, *φ* = 0.21) (Fig. 4B). Specifically, participants were more likely to report the familiar item in the final three positions than in the first three positions (57.1%, binomial test, *p* < 0.0001). To further investigate why participants would report the familiar item “out of order” with respect to precision, we tested whether the position the familiar item was reported influenced the response errors for novel items. We controlled for the effect of response order on precision by only using responses 2–5 where both a before and after response could occur. For example, a novel item reported second would be considered *after* if the familiar item was reported first on that trial, whereas it would be considered *before* if the familiar item was reported third or later. There was a consistent benefit in reporting novel items before the familiar item as compared to reporting them after the familiar item (*t*(61) = 11.28, *p* < 0.0001, *d* = 1.60) and this was true for all non-terminal response positions except response 3 that did not survive Bonferroni correction (response 2: *t*(59) = 2.88, *p*_*(Bon)*_ = 0.0056, *d* = 0.50, *α* = 0.013 ; response 3: *t*(61) = 2.47, *p*_*(Bon)*_ = 0.016, *d* = 0.35, *α* = 0.013; response 4: *t*(60) = 4.98, *p*_*(Bon)*_ < 0.0001, *d* = 0.64, *α* = 0.013; response 5: *t*(60) = 6.08, *p*_*(Bon)*_ < 0.0001, *d* = 0.88, *α* = 0.013) (Fig. 4B). These results did have a significant effect of experiment group (*F*(1, 60) = 5.48, p = 0.023, η_p_^2^ = 0.08).

### Precision depends on the distance from the prior location

**Figure 5:**
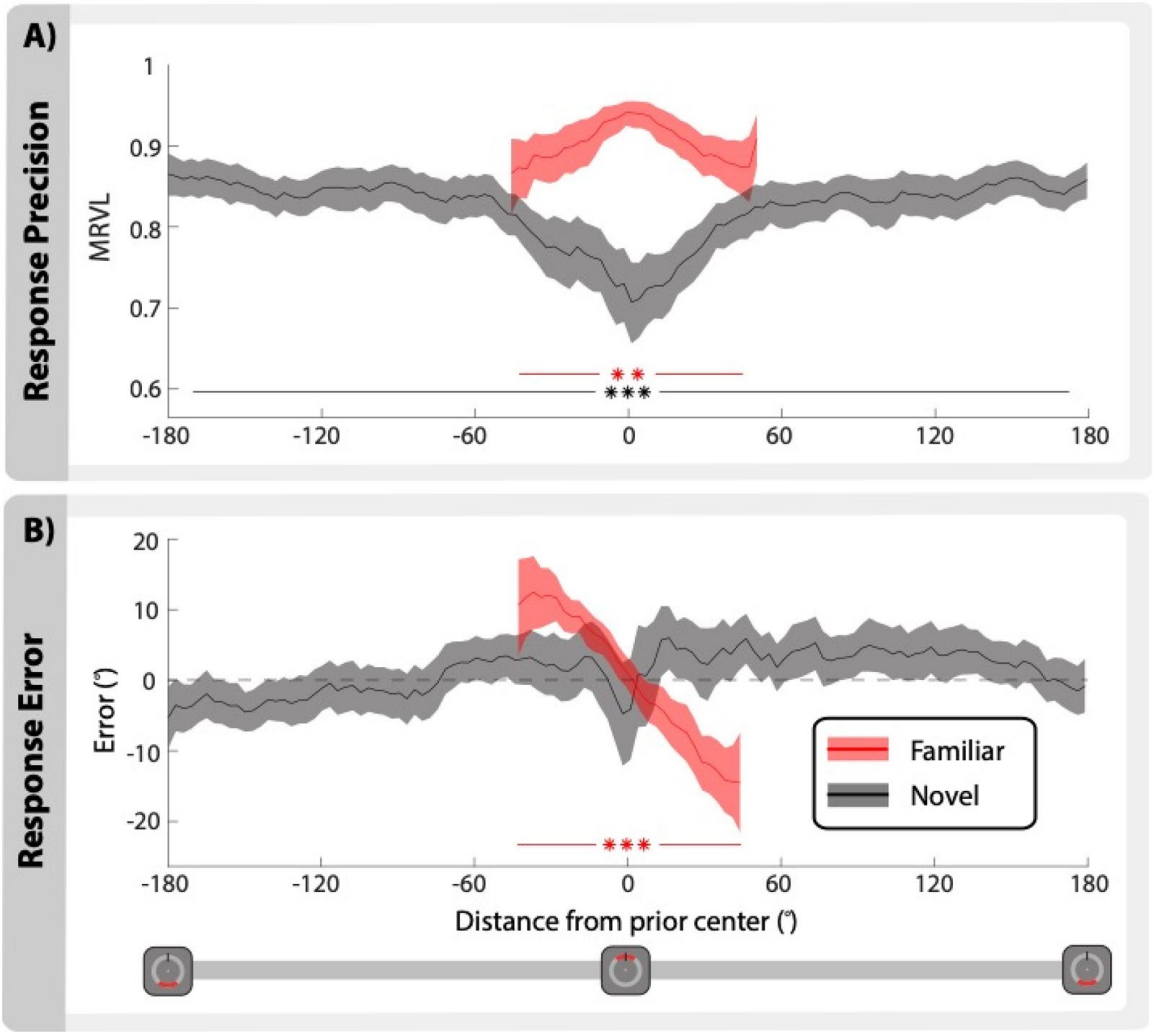
Attentional bias away from the prior location. (**A**) Precision and (**B**) error for novel and familiar items as a function of location, where zero marks the center of the prior location. Error is equal to the response minus the true location, where a negative slope indicates a bias towards the prior and a positive slope indicates a bias away from the prior. Familiar items did not appear outside the prior location distribution. Solid lines show the means, and the shaded areas indicate bootstrapped 95% CIs (ANOVA ** *p* < 0.01, *** *p* < 0.0001).

We tested whether precision for both the familiar and novel items was influenced by where each item appeared on the screen. Location was centered relative to the mean of the prior. Note that the familiar item did not appear outside of the prior distribution, therefore no responses are shown for the familiar item outside of this range. Using separate one-way ANOVAs we found location to influence precision for both the familiar item (*F*(9, 441) = 2.52, *p* = 0.0051, η_p_^2^ = 0.05) and novel items (*F*(35, 2135) = 3.72, *p* < 0.0001, η_p_^2^ = 0.06). Precision was separated into two bins representing the center (−10° to 10°) and the surrounding locations (− 180° to − 9°, 11° to 180°). Novel items that appeared near the center of the prior location had worse precision than anywhere else (*t*(61) = 4.54, *p* < 0.0001, *d* = 0.78). Conversely, the precision for the familiar item increased when it appeared near the center of the prior (*t*(61) = 10.17, *p* < 0.0001, *d* = 1.08) (Fig. 5A). The improvement in precision at the center of the prior can be attributed to a systematic response bias towards the center of the distribution. This was shown through an influence of location on the response errors (*F*(9, 441) = 12.72, *p* < 0.0001, η_p_^2^ = 0.20), where responses were 3.2° closer to the mean for every 10° bin away from the mean (*β* = -3.22, *p* < 0.0001, *R*^2^_*(adj)*_ = 0.16) (Fig. 5B). Similarly, it is possible that the reduction in precision at the center of the prior for the novel items could also be due to a response bias. However, the response errors for these items show no influence of location (*F*(9, 540) = 1.10, *p* = 0.36, η_p_^2^ = 0.02). These results had no main effect of experiment group (*F*(1, 60) = 0.50, *p* = 0.48, η_p_^2^ = 0.01) or location (*F*(9, 540) = 1.14, *p* = 0.33, η_p_^2^ = 0.02). However, there was an interaction (*F*(9, 540) = 3.29, *p* = 0. 0006, η_p_^2^ = 0.05), where only experiment 2 showed a main effect for location (*F*(9, 198) = 2.54, *p* = 0.0088, η_p_^2^ = 0.10).

## Discussion

In this study we showed that response precision for visual stimuli in a full-report WM task increases when participants have access to prior information about those stimuli. Specifically, when the probable location of one item in a set of six items is known prior to encoding, the precision with which the locations of both the familiar item and the other five items on each trial can be reported after a brief delay is increased (Fig. 2). This demonstrates that participants can and do use relevant LTM information to minimize errors and enhance the effective capacity of WM. Participants benefited from this information by using it to report the location of the familiar item rather than encoding that item into WM on every trial. This allowed for their capacity-limited WM resources to be allocated strategically to encode novel items that did not have prior information. This benefit was observed in the response characteristics from a full-report procedure where participants were forced to respond to each item. Responses for novel items were systematically ordered such that participants reported the items in descending order of precision (Fig. 3). This replicates the results observed in Adam et al., 2017^31^, in which the authors demonstrated that such ordering was not due to output interference^32^ but derived from the variable precisions of the item representations in WM. This precision-dependent response ordering was also observed for the familiar item in our study. However, the precision of familiar items reported in the final three responses plateaued and was greater than the precision of the novel items. This result aligns with the proposed strategy where subjects rely on prior information to report the familiar item rather than encoding it into WM.

It should be noted that this precision-dependent ordering was only found when evaluating each item type independently. Within trials, where participants reported both the familiar item and the novel items, this ordering effect was interrupted by a report of the familiar item (Fig. 4A). In other words, the order in which the familiar item was reported cannot be explained by the response precision alone. Given the systematic negative relationship found between precision and response order for novel items, it is possible that when participants prioritized the novel items, they may have reported the familiar item later in the response sequence. According to the data shown in Fig. 3B, prioritizing novel items could be a beneficial strategy because unprioritized novel items have significantly worse precision than unprioritized familiar items across the final three response positions. Indeed, we found the response order of the familiar item was biased towards the latter half of responses (Fig. 4B), although nearly all participants reported the familiar item at every response position across trials. This suggests that on some trials the participants employed a suboptimal strategy and reported the familiar item *before* some or all of the novel items. We hypothesize that this behavior is a reflection of WM’s limited capacity where participants waited to report the familiar item after first reporting other high-precision memory representations. For example, on any given trial, a participant would encode items with variable amounts of precision in WM^3,4^. Participants would report the items with the greatest precision first, followed by an estimate of the familiar item based on the prior, and conclude with “best guesses” for any remaining items that are stored with little to no precision in WM. According to this procedure, the response to the familiar item could be marking a *boundary* between responses with high and low precision. This could be a descriptive feature of the data, or a deliberate strategy used by participants. Consistent with this idea, we found that novel items that were reported before the familiar item had higher precision than novel items reported after the familiar item (Fig. 4C). Of note, these results can also align with recent work arguing against a differentiation between in-memory responses and guesses and instead asserts all responses derive from a single process governed by psychological similarity^33^. In this case, the demarcation may be made by participants’ metacognition rather than a differentiation between degrees of precision of memory representations. We did not, however, collect confidence ratings in this study and so we cannot evaluate whether participants strategically ordered their responses by subjective confidence. The hypothesis that familiar item responses strategically demarcate high and low confidence responses for novel items should be tested directly in future research.

Finally, an analysis of responses as a function of spatial location indicated that participants were able to prioritize novel items by narrowing their range of spatial attention during encoding. This attentional bias was evidenced by a decrease in precision for novel items that appeared near the center of the prior location (Fig. 5A). This reduction in precision was not due to a response bias, i.e., participants were not *less likely* to report a novel item as having appeared in the prior distribution, they were simply *less accurate* at doing so. These results are consistent with an interpretation that attention was deprioritized for this location during encoding, leading participants to not precisely encode the location of the familiar item from trial to trial. Without a precise representation of the familiar item in WM, participants used the prior information to bias their responses towards the mean of the prior, i.e. the most probable location of the familiar item. This strategy allowed participants to reduce the error for familiar items without using additional WM resources. Based on these results, we infer that the increase in precision for novel items following the introduction of the location prior was due to participants’ strategic prioritization for encoding these items by allocating attention *away* from the prior. This demonstrates that the incorporation of LTM information improved strategic resource allocation and WM performance.

The present results may be limited to situations where the statistics of the environment are well known. Participants knew the familiar item would appear on every trial, allowing them to potentially use the same strategy for every trial. If participants did not know whether the familiar item would appear in the following trial, they may find it difficult to use the LTM information in the same way. Previous work has shown people are capable of using previous experience in WM tasks using memory-guided attention^28,34^, but adequate time may be necessary to allow participants to optimally attend to their environment. Participants may also rely on LTM less as the prior information becomes less reliable.

Additionally, these findings may, in fact, result from a compression process^19,20^ in WM. For example, familiar items may not require the same amount of WM resources to achieve the same level of response precision for novel items that were prioritized at encoding. Participants could possibly do this by encoding the item location as a compressed label, such as “*slightly clockwise of the mean location*” rather than encoding a precise spatial location. This would likely require more time to recode and report the item. Unfortunately, the full-report paradigm does not allow for a meaningful collection of reaction times to evaluate this possibility, but this is a useful question for future research. Although previous studies have shown statistical regularities can be implicitly learned^35–37^ the use of such a compression strategy may only be possible if participants are consciously aware of the prior information. Ngiam et al., (2019)^38^ showed that compression only occurred when participants were aware of the statistical regularities, thus implicit learning of the prior information may not produce the same results. Of note, combining bottom-up information with prior information, such as in Bayesian learning, or combinations of learned statistical regularities (priors) with incoming sensory information (likelihood) to form the perceived stimuli (posterior), is possible for perception^39,40^. The use of implicit prior information may, however, be restricted to perception and not extend to information held in WM.

Previous work has shown that people can use behavioral relevance or uncertainty estimates to inform WM allocation decisions^5,16^. Here, we have shown that people can also incorporate prior knowledge about stimuli in determining how to allocate limited WM resources when faced with a memory challenge beyond their capacity. Prior knowledge about an item guides attention away from familiar items and towards novel items, thus increasing the overall precision with which memory items can be reported. These results support the concept of WM as involving a dynamic interaction between new information in the environment and prior information in LTM.

## Methods

### Data and code availability

All data and code will be available in Open Science Framework.

### Participants

62 subjects completed the 60- to 90-min experiment (41 female; ages 18–21). All participants had normal or corrected-to-normal vision, were not colorblind, provided informed consent, and received either course credit or monetary compensation (6 subjects were paid $12/h). The study was conducted in accordance with the relevant guidelines and regulations in the Declaration of Helsinki and was approved by the Institutional Review Board (IRB) for Social Behavioral research at the University of Texas at Austin (IRB protocol #2013-10-0110).

### Stimuli

The experiment was written in Python using Psychopy and run on a 21.5in iMac screen (1920 × 1080 resolution and 60-Hz refresh rate). Participants viewed the screen from a distance of about 60 cm. Stimuli were shown on a gray background and a fixation point with a radius of 0.1°. Colored dots with a radius of 0.3° appeared at a random angle with a fixed eccentricity of 3.25°. Colored dots were shown in HSV with saturation and value equal to one. Only six colors were used and shown on every trial: red, orange, yellow, green, cyan, and blue (hue = 0°, 30°, 60°,120°, 180°, 240°, respectively). The locations of five of the six colors were determined by a uniform distribution (novel items), while the location for the remaining color was determined by a von Mises distribution with a standard deviation of 20° (familiar item). Additionally, a minimum separation of 30° was maintained between each item on the screen. The color of the familiar item (one of six colors) and mean of the location distribution (1 of 360 locations) were randomly determined for each participant and remained constant throughout the experiment. A ring of random noise from 2.76–4.5° eccentricity was used for the visual mask. During the response period, six colored dots of equal size to the ones shown during encoding appeared at the bottom of the screen in random order (5° below the center fixation and 1°, 3°, and 5° to the right and left of the center fixation) to facilitate the full-report procedure. To respond, participants first clicked on one of these dots and then indicated on the response ring the location at which that color had appeared in the stimulus display. Finally, feedback was given in two ways: (1) both the true locations and the responses of all six stimuli were shown using a filled and outlined colored dot, respectively, and (2) the points earned for each response were shown numerically by colored thermometers (size = 0.5° × 1.5°) at the bottom of the screen, where the height indicated the number of points earned for that color (see Fig. 1).

### Task

We used a full-report delayed-estimation task with a single continuous dimension (spatial location along a circle). Participants were tasked to remember and report the location of all six colored dots shown on the screen. On each trial, participants were shown six colored dots for 1,000 ms. After a visual mask (200 ms) and delay period (750 ms), participants would report the location of all six dots in any order. At the end of each trial participants were given feedback including the points earned for each response. Responses earned points as a normal function (M = 0°, SD = 30°) of error, where 10 points was the maximum for each response. Participants completed a block of trials once a point goal was achieved. The point goals were calculated in real time based on performance in the practice trials so that each block consisted of about 20 trials. Participants completed 8 blocks total with varying trial counts for each block (M = 18.43, SD = 2.38).

Before the main task, participants were informed of the color and location of their familiar item by a colored arc spanning two standard deviations from the center (total 80°). Participants then completed practice trials until a point goal was achieved (about 3 trials). During the practice trials the same colored arc would appear when the participant was reporting the familiar item as a reminder. Upon completion of the practice trials, participants were tested on their knowledge of the prior information. If participants could not correctly report from memory both the color and location (within the 80° arc) they would repeat another practice trial before being tested again. Once the participant passed, they would move on to the main task. Critically, this was the only time participants were shown the prior information and it remained constant throughout the experiment.

In order to collect a baseline response precision without prior information, Participants in experiment 1 (n = 39) were not shown the prior information until the second block of trials. All items in the first block thus were novel items. After the first block, participants were shown the prior information and tested in the same manner as the other participants. Additionally, these participants were asked to report the color and location of the prior information, same as the test, at the end of each block of trials.

### Analyses

#### General

All statistical values were calculated using standard parametric tests using the Pingouin^41^ statistics package in python. All t-tests were two-sided, and all post-hoc comparisons were Bonferroni corrected. Response precision was estimated using mean resultant vector length (MRVL, “circ_r.m”^42^). Some participants (n = 5 of 62) did not report the familiar item at every response number. Therefore, degrees of freedom changed slightly between tests isolating familiar items for each response number (i.e. Figure 3). Additionally, all confidence intervals shown in plots were calculated using bootstrapping methods where subjects were randomly sampled with replacement and iterated 10,000 times. No effect of experiment group was found aside from two analyses mentioned in the results: novel items reported before and after the familiar item (Fig. 4C) and response bias for novel items (Fig. 5B). Therefore, all subjects across both experiments were used in all analyses aside from the prior benefit analysis from Fig. 2.

#### Benefit from prior information (Fig. 2)

Three within subject t-tests were used to compare response precision with and without prior information for participants from experiment 1 (n = 39). Three different samples were used: without prior information and both the novel items and the familiar item with prior information. Precision without prior information was derived from all stimuli from the first block of trials. Precision with prior information was found separately for both familiar and novel items from the remaining seven blocks of trials.

To test for a practice effect, a within-subject t-test was used to compare response precision from the first block of trials to the remaining block of trials for participants from experiment 2 (n = 23). Finally, a planned between-subject t-test was used to control for the practice effect by comparing the precision from the first block of trials from experiment 1 (without prior information) and the first block of trials from experiment 2 (with prior information).

#### Precision by response number (Fig. 3)

A repeated measures two-way ANOVA was used to test for a main effect of response number and item type as well as an interaction between them. Post-hoc within-subject t-tests were used to compare response precision between familiar and novel items within each response number. Linear regression was additionally used to show the linear trend between precision and response number for both familiar and novel items.

#### Response order (Fig. 4)

Response order for the familiar item was compared to a uniform distribution using a chi square goodness of fit test. Frequency of familiar item responses was summed across all subjects for each response number. Number of trials per subject varied (M = 18.43, SD = 2.38). Responses were then binned in early (responses 1–3) or late (responses 4–6) groups, where frequency was compared using a binomial test.

Precision was compared between novel items reported before and after the familiar item using within-subject t-tests. Before and after responses were only compared within each response number to control for the effect of response number on precision.

#### Location bias (Fig. 5)

All locations were centered for each participant so that the mean of the von Mises distribution was equal to zero. A repeated measures one-way ANOVA was tested on the MRVL of 10° bins for both the familiar and novel items. Novel items spanned the full 360° range (36 bins), while the familiar item only spanned a range of 100° around the center (10 bins). Within-subject t-tests comparing the center bins (− 10° to 10°) to outer locations (− 180° to − 9°, 11° to 180°) were used for the follow-up comparisons. Finally, the plotted data were smoothed using a 15° sliding window with a step size of 3°.

To evaluate response bias, we used the response error to examine participants’ responses relative to where the familiar item was located on each trial. Similarly, repeated measures one-way ANOVAs were used to evaluate the influence of location on response errors. A follow-up linear regression was used to quantify the degree of bias for the familiar item.
